# Fine Wrinkle Improvement through Bioactive Materials That Modulate *EDAR* and *BNC2* Gene Expression

**DOI:** 10.3390/biom14030279

**Published:** 2024-02-26

**Authors:** Seonju Lee, Sanghyun Ye, Mina Kim, Hyejin Lee, Seung-Hyun Jun, Nae-Gyu Kang

**Affiliations:** LG Household and Health Care, R & D Center, Seoul 07795, Republic of Korea; seonju@lghnh.com (S.L.); shye123@lghnh.com (S.Y.); mnkim@lghnh.com (M.K.); hellohj1223@lghnh.com (H.L.)

**Keywords:** skin aging, fine wrinkle, wrinkle improvement, cosmetic ingredients, GWAS

## Abstract

Skin aging is a multifaceted biological phenomenon influenced by a combination of intrinsic or extrinsic factors. There is an increasing interest in anti-aging materials including components that improve skin wrinkles. Despite the availability of several such wrinkle-improving materials, the demand for ingredients with outstanding efficacy is increasing. Therefore, this study aimed to explore the mechanisms of wrinkle-related genes reported in previous genome-wide association studies (GWASs), identify materials that regulate these genes, and develop an effective anti-wrinkle formula containing the active ingredients that regulate the expression of these genes. We selected two candidate genes, *EDAR* and *BNC2*, that are reportedly related to periorbital wrinkles. We investigated their functions in the skin through in vitro experiments using human skin cell lines (keratinocytes and fibroblasts). Moreover, we identified ingredients that regulate the expression of these two genes and confirmed their efficacy through in vitro experiments using the skin cell lines. Finally, we developed a formula containing these ingredients and confirmed that it enhanced dermal collagen in the 3D skin and improved fine wrinkles under the eyes more effectively than retinol in humans, when applied for 8 weeks. Our results are significant and relevant, as we have discovered a special formula for wrinkle improvement with reliable efficacy that surpasses the efficacy of retinol and does not cause side-effects such as skin irritation.

## 1. Introduction

The skin is a protective barrier that isolates the inside of the human body from external environments. It consists of three layers: the epidermis, dermis, and subcutaneous tissue [1]. The epidermis, the outermost layers, protects against environmental pathogens and regulates skin hydration through trans-epidermal water loss [2].

The current society longs for healthy skin, and the pharmaceutical industry is developing various formulations, from cosmetics to dermatological treatments, to achieve this. Healthy skin represents a younger appearance than the actual physical age, not just good health and well-being. Therefore, various studies are being actively conducted to understand skin aging and find effective solutions to suppress it.

There are two major processes that cause skin aging: intrinsic and extrinsic. Intrinsic aging is a process of natural chronological change that reflects genetic characteristics in particular [3]. In addition, the biological effects of intrinsic aging alter the physiological state of the skin, leading to impaired and/or delayed wound healing [4]. Extrinsic aging is induced by environmental factors such as exposure to the sun—specifically, ultraviolet radiation, air pollution, and smoking [5]. Extrinsic aged skin is characterized by sunburn freckles, lentigines solaris, pigment change, coarse wrinkles, dryness, and elastosis, whereas intrinsic aged skin is characterized by uneven pigmentation, fine wrinkles, lax appearance, and reduced fat tissues [6,7,8]. In addition to the individual effects of intrinsic and extrinsic aging, the interaction between the two factors is also important to the skin. Previous studies have shown that environmental factors, such as pollutants, diet, and sun exposure affect epigenetic changes and gene mutations that impair skin conditions [9].

Among internal factors, genetic diversity (in other words, gene polymorphisms) is one of the major factors that determines the development of skin aging characteristics such as wrinkles and pigment spots. Recently, genome-wide association studies (GWASs) have been actively conducted as one of the approaches to determining the fundamental causes of skin aging. A recent Chinese GWAS [10] identified multiple loci associated with skin aging-related characteristics such as crow’s feet, periorbital wrinkles, and nasolabial fold. A Latin American study revealed new loci associated with skin wrinkles around the forehead, under the eye, and around the glabella [11]. A European study reported multiple loci related to age perception [12]; in particular, *MC1R*, reported in this study, was also identified in the aforementioned study on a Latin American population. Kim et al. [13] identified single-nucleotide polymorphisms (SNPs) for wrinkles, pigmentation, moisture, oil, and sensitivity. Thus, several studies on wrinkles as well as other skin characteristics have been conducted.

Notably, the most representative phenomena of aging is wrinkles, and wrinkles, especially around the eyes, are the first to occur during aging [14,15]. The early occurrence of wrinkles near the eyes is attributed to the fact that the periorbital skin is the thinnest facial skin, experiences constant movement caused by blinking, has reduced subcutaneous fat, and is exposed to ultraviolet rays [14,16]. As wrinkles around the eyes reportedly have a significant influence on the perception of age [17,18,19], they should be treated well for a healthier and younger appearance. SNPs related to the periorbital skin, the location for under-eye wrinkles and crow’s feet, have been identified by a GWAS [20]. This study identified seven genetic loci significantly associated with under-eye wrinkles: rs13005242 (500 bp downstream of *EN1*), rs4894405 (3q23), rs12503455 (intron in *SPATA5*), rs117927610 (4q31.21), rs10117511 (9p21.3), rs749810 (intron in *EDAR*), and rs74653330 (exon in *OCA2*) [20]. In addition, six genetic loci associated with crow’s feet were discovered: rs10810635 (intron in *BNC2*), rs72620727 (12q21.33), rs74653330 (exon in *OCA2*), rs2240751 (exon in *MFSD12*), rs36015125 (intron in *FARSB*), and rs11198112 (10q26.11) [20]. Although this study identified genes related to various wrinkles, it has a limitation: it did not investigate the mechanisms of genes in relation to wrinkles.

The ectodysplasin A receptor (*EDAR*) gene encodes proteins required for the formation of ectoderm. EDAR stimulation activates a series of downstream signals, such as the receptor adapter EDAR-related death domain (EDARADD) and nuclear factor kappa light chain enhancer of activated B cells (NF-κB) [21]. Specially, the EDAR pathway upregulates NF-κB transcription factors that promote wound healing via their anti-inflammatory and anti-oxidant effects [22]. Recently, EDAR signaling was found to play an important role in adult skin wound healing; mice lacking the EDA ligand exhibited a reduced wound-healing ability, whereas enhancing EDAR signaling promoted wound healing, even in wild-type mice [23]. EDAR pathway manipulation affects several aspects of the wound-healing process, including extracellular matrix remodeling and collagen deposition, and EDAR reinforcement promoted human skin wound healing [23]. The relationship between wound healing and skin aging deserves consideration; many works of research reported delayed or impaired wound healing in the aging process [4]. These age-related alterations in wound healing were suspected due to increased fibroblast senescence and the delayed proliferation and migration of keratinocyte and fibroblast. For this reason, some studies have verified that the promotion of wound healing refers to the improvement of the proliferation and migration of skin cells, which might finally beneficially affect wrinkle alleviation [24,25]. Therefore, we hypothesized that EDAR would be involved in wrinkles through a wound-healing mechanism.

Basonuclin2 (BNC2), a member of the Basonuclin zinc-finger family of transcription factors, has a nuclear localization signal and plays a role in regulating gene expression in germ cells and skin keratinocytes [26]. *BNC2* expression is reportedly related to cell survival in various cancer cells [27,28]. The BNC2 level was reduced in high-grade serous ovarian cancer samples compared to that in control samples, and the reduction in BNC2 levels increased cell survival after H_2_O_2_ treatment [27]. Overall, *BNC2* overexpression causes cell death in reactive oxygen species (ROS)-induced cells. Thus, BNC2 potentially affects cell survival after an oxidative stress response. Previous studies showed that oxidative stress is generated by UV exposure and that oxidative stress is highly associated with photoaging [29,30]. Therefore, controlling oxidative stress by regulating *BNC2* may be a possible target for helping the skin condition and aging.

In the present study, we explored the mechanisms underlying the action of *EDAR* and *BNC2*, which are associated with facial fine wrinkles represented by under-eye wrinkles and crow’s feet. We hypothesized that a decrease in the EDAR level in skin cells would result in a decreased wound-healing efficacy, while a reduced BNC level would protect against cell death through the anti-oxidant system. Moreover, we aimed to develop an effective anti-wrinkle formula that acts by regulating the expression of these genes. Through screening, we found materials that can regulate *EDAR* and *BNC2* and evaluated their efficacy in regulating the expression of genes related to wound healing and the anti-oxidant system as well as their ability to improve skin wrinkles. We confirmed the efficacy of *EDAR*- and *BNC2*-regulating materials through various in vitro experiments, developed a formula comprising these materials, and validated its efficacy in wrinkle improvement in humans.

## 2. Materials and Methods

### 2.1. Cell Culture

Human skin keratinocytes HaCaT (AddexBio, San Diego, CA, USA) and human skin fibroblasts Hs68 (American Type Culture Collection, Manassas, VA, USA) were used to assess the efficacy of active ingredients (Table 1). Hs68 were cultured in DMEM (Gibco, Grand Island, NY, USA) supplemented with 10% FBS (Gibco) and penicillin-streptomycin (Gibco) at 37 °C with 5% CO_2_. HaCaT were cultured in DMEM supplemented with 10% FBS, penicillin-streptomycin, 1 mM sodium pyruvate (Gibco), 2 mM L-glutamine (Gibco), and 0.01 mM CaCl_2_ (Sigma-Aldrich, St Louis, MO, USA) at 37 °C with 5% CO_2_.

### 2.2. Transfection of Keratinocytes and Fibroblasts with siRNA Duplexes for RNAi Experiments

HaCaT and Hs68 were cultured for 24 h in six-well plates to ensure complete attachment. HaCaT and Hs68 cells were seeded into six-well plates at a density of 1.5 × 10^5^ and 3 × 10^5^ cells/well, respectively, and allowed to reach 70–80% confluence. They were then transiently transfected with 100 nM *EDAR* and *BNC2*-specific siRNA duplexes (Bioneer, Daejeon, Republic of Korea) using Lipofectamine RNAiMax (Invitrogen, Carlsbad, CA, USA), following the manufacturer’s instructions. After transfection for 6 h, the reaction mixture was removed, the cells were washed, and the culture medium was replaced with fresh DMEM containing 10% FBS. After transfection for 36 h, the cells were harvested for analysis. The interference efficiencies of *EDAR* and *BNC2* siRNAs were measured by RT-qPCR.

### 2.3. RT-qPCR

HaCaT and Hs68 were seeded into six-well plates at a density of 2 × 10^5^ and 5 × 10^5^ cells/well, respectively, and incubated for 24 h at 37 °C. Then, the cells were treated with the active ingredients at various concentrations for another 24 h in serum-free medium. Following incubation, RNA was extracted from HaCaT and Hs68 cells using the RNeasy mini kit (Qiagen, Hilden, Germany). The concentration and purity of the extracted RNA were confirmed using a Nanodrop (Thermofisher, Wilmington, DE, USA). Total RNA (1 μg) was reverse-transcribed to cDNA using a cDNA synthesis kit (Philekorea, Seoul, Republic of Korea), following the manufacturer’s protocol, and a Veriti 96-Well Thermal Cycler (Applied Biosystems, Foster City, CA, USA). The following conditions were applied for reverse transcription: 42 °C for 30 min and 72 °C for 10 min. cDNA was amplified using TaqMan™ Universal PCR Master Mix (Applied Biosystems), according to the protocol by manufacturer, through the StepOnePlus^TM^ RT-PCR system (Applied Biosystems). The PCR reaction conditions were as follows: 30 cycles at 95 °C for 45 s, 60 °C for 1 min, and 72 °C for 45 s. qPCR was performed using the following commercial Taqman primers (Thermofisher): *EDAR* (Hs00223468_m1); *BNC2* (Hs00417700_m1); *MMP1* (Hs00899658_m1); *COL4A1* (Hs00266237_m1); *AQP3* (Hs01105469_g1); *IVL* (Hs00846307_s1); *COL1A1* (Hs00164004_m1); *HAS3* (Hs00193436_m1); *LAMA3* (Hs00165042_m1); *ITGA6* (Hs01041011_m1); *FGF10* (Hs01045105_m1); *FGF2* (Hs04187682_g1); *TGFβ* (Hs00998133_m1); CAT (Hs00937395_m1); *GPX1* (Hs01028922_g1); and *SOD1* (Hs00166575_m1).

### 2.4. Cell Migration (Scratch Assay)

HaCaT cells were seeded into 24-well plates at a density of 4 × 10^4^ cells/well and cultured for 24 h to reach 95–100% confluence. At that point, and before changing the medium, a scratch was made in the center of each well using a sterile 200 μL pipette tip to “injure” the HaCaT monolayer. After introducing the injury, the wells were washed twice with 1 mL PBS before imaging and treating with active ingredients. The plates were incubated for the next 2 days until the scratch wound completely closed. Each well was photographed using an Olympus CKX41 light microscope (Olympus Corporation, Tokyo, Japan) with a 10× objective to capture the wound area on days 0, 1, and 2. Scratch area images were quantified using Image J software (NIH Image, Bethesda, MD, USA). The wound area in each well was normalized to that at the initial time-point and expressed as a percentage of wound closure.

### 2.5. Cell Viability (CCK-8 Assay)

Hs68 cells were seeded into 96-well plates at a density of 5 × 10^3^ cells/well and cultured until 70–80% confluence. Then, the cells were treated with each active ingredient at different concentrations as well as siRNA. Thereafter, 500 μM or 1000 μM H_2_O_2_ was added to induce cell death. After 24 h of incubation, the cell viability was determined using a Cell Counting Kit-8 (CCK-8) cell proliferation assay (Dojindo Molecular Technologies Inc., Rockville, MD, USA), according to the manufacturer’s instructions.

### 2.6. 3D Skin Experiment

3D skin Neoderm^®^ was purchased from Tego Science (Tego Science, Seoul, Republic of Korea). 3D skin was maintained and cultured according to the manufacturer’s instructions. For the experiment, the test formulation, containing phloretin (0.05%), oryzanol (0.1%), sucralfate (0.15%), and lupeol (0.135%), was topically applied. For the control, the same formulation without the active materials mentioned above was used. The 3D skin was cultured for 2 days and then stained by Masson’s trichrome. The sample was photographed using an Olympus CKX41 light microscope (Olympus Corporation, Tokyo, Japan) and analyzed using Image J software version 1.54 (NIH Image, Bethesda, MD, USA).

### 2.7. Human Clinical Trial

This study was approved by the Ethics committee of the LG H & H Institutional Review Board (LGHH-20211014-AA-02-01). Twelve healthy Korean volunteers (6 males and 6 females; age range, 28–48 years; mean age ± SEM, 33.167 ± 1.542 years) were recruited for the clinical trial. Pregnant women or those receiving skin treatment at the clinic were excluded. The test was performed in a half-face and double-blind manner. The test formulation, containing phloretin (0.05%), oryzanol (0.1%), sucralfate (0.15%), and lupeol (0.135%), was topically applied on the right side of the face twice daily for 8 weeks. As a control, 0.1% retinol formulation without the active ingredients mentioned above was applied on the left side of the face. Before facial wrinkle measurements, all participants were asked to rest for a minimum of 20 min in a humidity- (45 ± 5%) and temperature-controlled (22 ± 2 °C) room after cleaning their face. Under-eye wrinkles were measured in triplicate using a topographic skin measurement device (Antera 3D CS (Mravex, Dublin, Ireland)) every 4 weeks. 3D skin images were captured at the beginning of the experiment and after 8 weeks and analyzed to determine the changes in facial wrinkles.

### 2.8. Statistical Analysis

The data are presented as the mean and standard error of the mean (SEM) of at least three independent experiments. Statistical comparisons were conducted using the student *t*-test. Differences were considered as statistically significant at *p* < 0.05, *p* < 0.01, and *p* < 0.001. The data were analyzed using GraphPad Prism version 6.07 (GraphPad Software, La Jolla, CA, USA).

## 3. Results and Discussion

### 3.1. In Vitro Functional Study on Periorbital Skin Wrinkle-Related Genes, EDAR and BNC2

#### 3.1.1. Prevention of HaCaT Cell Migration and Wound Healing-Related Gene Expression by EDAR Knockdown

Previous reports suggest that wound repair is impaired by the aging process and that approaches to controlling the age-related wound healing are necessary for anti-aging strategies [31]. As EDAR activation reportedly promotes skin repair, we investigated its involvement in the skin wound-healing and -repair process.

**Figure 1:**
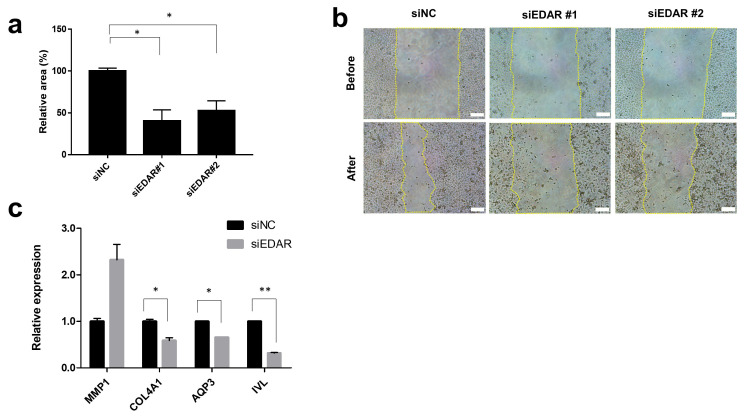
Effects of *EDAR* knockdown in skin cells. (**a**) Wound-healing efficiency after *EDAR* knockdown in keratinocytes (HaCaT). (**b**) Representative image from in vitro scratch wound-healing assay; scale bar = 146 μm. Yellow lines represent the wound boundary. (**c**) Expression of genes related to wound healing in *EDAR* knockdown cells. Error bars represent the standard error of the mean. * *p* < 0.05, ** *p* < 0.01; student *t*-test.

We selected the spontaneously immortalized HaCaT cell line for our in vitro experiments because it is a widely employed keratinocyte model owing to its ease of propagation and near-normal phenotype [32]. We transfected HaCaT cells with siRNA to specifically suppress *EDAR* expression and observed the effect of *EDAR* knockdown on cell migration, which is a measure of wound-healing efficacy. First, we confirmed the reduced *EDAR* expression in si*EDAR*-transfected cells compared to that in control siRNA (siNC)-transfected cells. Next, a scratch assay using si*EDAR*-treated cells confirmed that their wound-healing efficacy was significantly reduced compared to that of siNC-treated cells (Figure 1a,b). This finding was consistent with our hypothesis, which was based on the previous report [23]: a decrease skin cell EDAR levels will reduce the wound-healing efficacy.

Next, we measured the expression of genes associated with wound healing in cells with reduced *EDAR* expression and wound-healing efficacy. We observed an increased expression of *MMP1* and a decreased expression of *COL4A1*, *AQP3*, and *IVL* in siEDAR-treated cells that exhibited decreased *EDAR* expression (Figure 1c). *MMP1* encodes proteins involved in cellular collagen degradation and plays a role in the wound-healing process [33]. The excessive expression of *MMP1* can impair the wound-healing process [33]. *COL4A1* encodes proteins residing in the cell basement membrane and was found to be involved in keratinocyte proliferation and the formation of an epidermal layer [34]. *AQP3* plays an essential role in various processes involved in keratinocyte function, especially water transportation [35]. *APQ3* expression is strongly related to skin epidermal homeostasis and wound healing [36]. *IVL* encodes involucrin, which is essential for skin barrier formation and integrity [37]. Thus, our findings suggest that the reduction in *EDAR* expression can lead to the impairment of wound healing and that controlling *EDAR* expression can be a potential wound-healing strategy.

#### 3.1.2. Increase in Cell Survival and Skin Extracellular Matrix-Related Gene Expression by BNC2 Knockdown

Apoptosis or cell death is one of the causes of aging that must be overcome for anti-aging strategies. Cells accumulate damage as they age, which eventually leads to cell senescence and, subsequently, cell death [38]. Therefore, the activation of an intracellular antioxidant system to prevent cell damage caused by various environmental factors such as UV and ROS is worth exploring as an anti-aging strategy, and various cosmetic ingredients are already being developed to target this mechanism [39]. As the reduction in *BNC2* expression increased cell survival, we hypothesized that *BNC2* knockdown, resulting in reduced *BNC2* expression in skin cells, may protect cells from oxidative stress-induced apoptosis.

**Figure 2:**
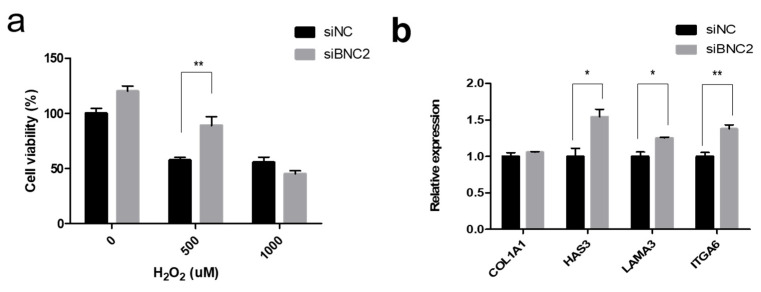
Effects of *BNC2* knockdown in skin cells. (**a**) Cell viability of H_2_O_2_-treated fibroblasts (Hs68) with or without *BNC2* knockdown. (**b**) Expression of genes related to the extracellular matrix in *BNC2* knockdown cells. Error bars represent the standard error of the mean. * *p* < 0.05, ** *p* < 0.01; student *t*-test.

First, we confirmed the active expression of *BNC2* in skin fibroblasts. Thereafter, we confirmed that the expression of *BNC2* decreased in si*BNC2*-treated Hs68, a fibroblast cell that was isolated from the foreskin. Then, we evaluated the survival of *BNC2*-silenced cells after hydrogen peroxide (H_2_O_2_) treatment. As expected, the cell death level was lower in the si*BNC2*-treated group compared to that in the control siNC-treated group (Figure 2a). However, treatment with ≥1000 μM H_2_O_2_ resulted in similar levels of cell death regardless of the *BNC2* expression level. Our findings suggest that *BNC2* expression does not have a significant effect on cell death in situations of excessive oxidative stress. Since aging is a phenomenon that progresses due to the long-term accumulation of oxidative stress [40], the regulation of *BNC2* expression can be used as an anti-aging strategy. Moreover, the removal of excessively damaged cells is important to maintain homeostasis and block cancer development. Our results showed that treatment with a high concentration of H_2_O_2_ resulted in similar levels of cell death regardless of the *BNC2* expression level. Therefore, these results suggest that reduced *BNC2* expression will not inhibit the removal of excessively damaged cells.

To evaluate whether decreasing *BNC2* expression has potential as an anti-wrinkle strategy, we analyzed changes in the expression levels of genes related to the extracellular matrix in the skin. In cells with reduced *BNC2* expression due to *BNC2* silencing, we observed a significant increase in the expression of *HAS3*, *LAMA3*, and *ITGA6* along with cell survival (Figure 2b). Collagen is one of the main components of the skin and plays an important role in imparting connective tissue elasticity [41]. *COL1A1* encodes for Type I collagen, which is the most common type found in the skin, and is associated with skin wrinkles and elasticity [3,42]. *HAS3* encodes proteins involved in the synthesis of hyaluronic acid, a major component of the extracellular matrix [43]. *LAMA3* and *ITGA6* encode proteins that exist in the basement membrane of the skin and are involved in communication between the epidermis and dermis [44]. Based on these reports and our results, it can be inferred that a decrease in *BNC2* expression can lead to an increase in the hyaluronic acid and collagen content in the dermis and can exert an anti-aging effect in the skin by promoting communication between the epidermis and dermis.

### 3.2. Screening of EDAR and BNC2 Expression-Regulating Materials

After confirming that increasing *EDAR* expression and decreasing *BNC2* expression have potential as good wound-healing and anti-oxidant strategies, respectively, we screened materials to identify ingredients that regulate the expression of *EDAR* and *BNC2*.

**Figure 3:**
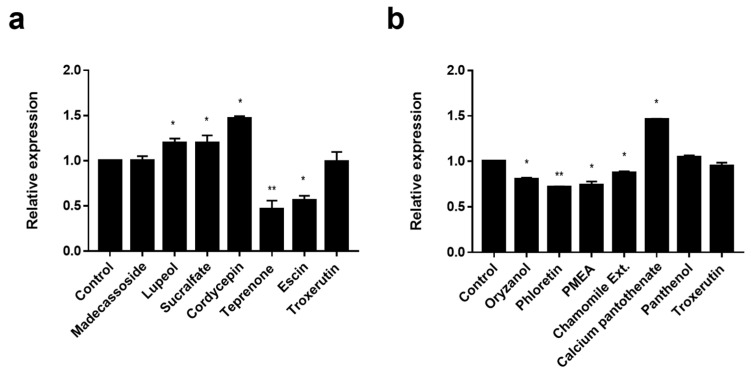
The screening of *EDAR* and *BNC2* expression-regulating materials. (**a**) The materials that increased the expression of *EDAR*. Relative expression of *EDAR* in keratinocytes (HaCaT) treated with various candidates. (**b**) The materials that decreased the expression of *BNC2*. Relative expression of *BNC2* in fibroblasts (Hs68) treated with various candidates. Error bars indicate the standard error of the mean. * *p* < 0.05, ** *p* < 0.01; student *t*-test.

Since EDAR is involved in the wound-healing pathway, we screened materials, such as madecassoside, that are known to have a wound-healing effect. Madecassoside is active in wound healing and acts through increasing anti-oxidative activity, enhancing collagen synthesis, and influencing angiogenesis [45]. However, we found a negligible effect on the expression of *EDAR* (Figure 3a). The materials that significantly increased *EDAR* expression in HaCaT cells after two days of treatment were lupeol (10 μg/mL, *p* < 0.05), sucralfate (10 μg/mL, *p* < 0.05), and cordycepin (0.1 μg/mL, *p* < 0.05) (Figure 3a). In addition, among the triterpenes similar to lupeol, teprenone (10 μg/mL, *p* < 0.01), escin (10 μg/mL, *p* < 0.05), and troxerutin (10 μg/mL) were found to inhibit *EDAR* expression (Figure 3a). Thus, the increase in *EDAR* expression was not due to the overall characteristics of wound-healing materials or triterpenes but to the specific action of lupeol, sucralfate, and cordycepin.

Since the knock-down of *BNC2* alleviated H_2_O_2_-induced cell death, we screened materials known to have anti-oxidant efficacy. Materials that decreased *BNC2* expression in Hs68 cells after two days of treatment were oryzanol (10 μg/mL, *p* < 0.05), phloretin (10 μg/mL, *p* < 0.01), palmitamide MEA (PMEA) (10 μg/mL, *p* < 0.05), and chamomile extract (1000 μg/mL, *p* < 0.05) (Figure 3b). Although calcium pantothenate (0.01 μg/mL, *p* < 0.05), panthenol (1 μg/mL), and troxerutin (1 μg/mL) possess anti-oxidant efficacy, they did not reduce the expression of *BNC2* (Figure 3b). Thus, it was found that the decrease in *BNC2* expression was not due to the overall characteristics of anti-oxidant materials but to the specific action of oryzanol, phloretin, PMEA, and chamomile extract.

In addition to the materials with wound-healing effects (for EDAR) or anti-oxidation effects (for BNC2), we further analyzed other substances reported to have wrinkle improvement effects, regardless of wound-healing or anti-oxidation effects, to determine their effect on *EDAR* and *BNC2* expression. We found that *EDAR* expression was enhanced by retinol (10 μM, *p* < 0.05), Matrixyl-3000 (Sederma, Le Perray en Yvelines, France; 1000 μg/mL, *p* < 0.05), Peptilium (Silab, Saint-Viance, France; 0.1 μg/mL, *p* < 0.001), and polydatin (0.5 μg/mL, *p* < 0.05), while BNC2 expression was reduced by Peptilium (Silab; 0.1 μg/mL, *p* < 0.05), bakuchiol (20 μM, *p* < 0.05), and Panax ginseng root protoplasts (PGRP; 100 μg/mL, *p* < 0.05) (Figure S1).

### 3.3. Wound-Healing and Anti-Oxidant Effect of EDAR and BNC2 Expression-Regulating Materials

#### 3.3.1. Wound-Healing Effect and Enhanced Expression of Wound Healing-Related Genes by EDAR-Upregulating Materials

**Figure 4:**
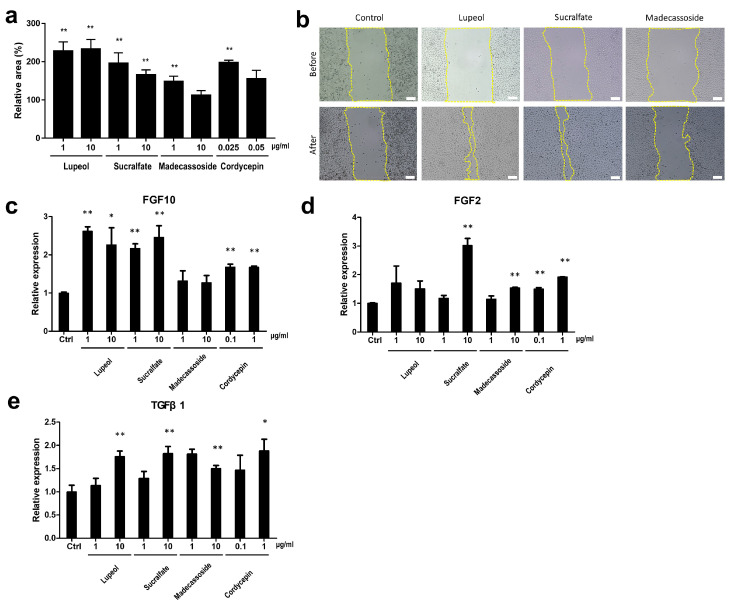
Analysis of the wound-healing effects of *EDAR* expression-regulating materials through in vitro experiments. (**a**) Wound-healing efficacy of *EDAR* expression-upregulating materials in HaCaT. (**b**) Representative image from the in vitro scratch wound-healing assay; scale bar = 122 μm. Yellow lines represent the wound boundary. (**c**) The enhancement of *FGF10* expression by *EDAR*-upregulating materials. (**d**) The enhancement of *FGF2* expression by *EDAR*-upregulating materials. (**e**) The enhancement of *TGFβ1* expression by *EDAR*-upregulating materials. Error bars represent the standard error of the mean. * *p* < 0.05, ** *p* < 0.01; student *t*-test.

Since the wound-healing efficacy reduces as the EDAR expression decreases, we conducted in vitro scratch assays to determine whether the materials that increase EDAR expression can promote actual wound healing. In the case of lupeol, sucralfate, and cordycepin treatment, which increased EDAR expression by more than 1.15 times (Figure 3a), the wound-healing efficacy was high; although madecassoside treatment increased wound healing, it was not significant compared to that by other materials (Figure 4a,b). In particular, treatment with a high concentration (10 μg/mL) of madecassoside did not increase EDAR expression nor demonstrate significant wound-healing efficacy (Figure 4a,b). Therefore, materials that upregulate EDAR expression promote wound-healing efficacy and can potentially be used as excellent skin-improving ingredients.

Wound healing is a complex process that involves several cell types and sequential repair responses in damaged tissue [46]. For example, the wound-healing process involves the activation of not only keratinocytes but also fibroblasts, and the wound is considered to be completely healed when the basement membrane regeneration is complete [47]. During wound healing, the migration of fibroblasts is activated by the basic fibroblast growth factor (bFGF) and transforming growth factor (TGF) [22]. Moreover, a study using mouse skin found that FGF10 plays an important role in the normal maintenance of basal cells, one of the main components of the basement membrane [48]. Thus, we tested the changes in the expression of *FGF10*, *FGF2*, and *TGFβ1* in fibroblasts and confirmed that treatment with lupeol, sucralfate, cordycepin, and madecassoside increased the expression of all three genes (Figure 4c–e). The expression of *FGF10* was increased by material treatment in the following order: lupeol (*p* < 0.01), sucralfate (*p* < 0.01), and cordycepin (*p* < 0.01), with lupeol and sucralfate being particularly effective (Figure 4c). Because several studies reported the role of madecassoside in fibroblasts [49,50], excellent efficacy was expected; however, we found that its treatment did not result in a statistically significant increase in gene expression (Figure 4c). *FGF2* expression was significantly promoted by sucralfate (*p* < 0.01), cordycepin (*p* < 0.01), and madecassoside (*p* < 0.01); although lupeol promoted the expression of *FGF2*, it was not statistically significant (Figure 4d). All four materials showed similar excellent efficacies in increasing the expression of *TGFβ1* (Figure 4e). Therefore, our results suggest that lupeol, sucralfate, and cordycepin are effective materials for targeting wound healing that can promote dermal and basement membrane regeneration in addition to epidermal regeneration.

#### 3.3.2. Cell Protection Effect and the Enhanced Expression of Anti-Oxidant System-Related Genes by *BNC2*-Downregulating Materials

**Figure 5:**
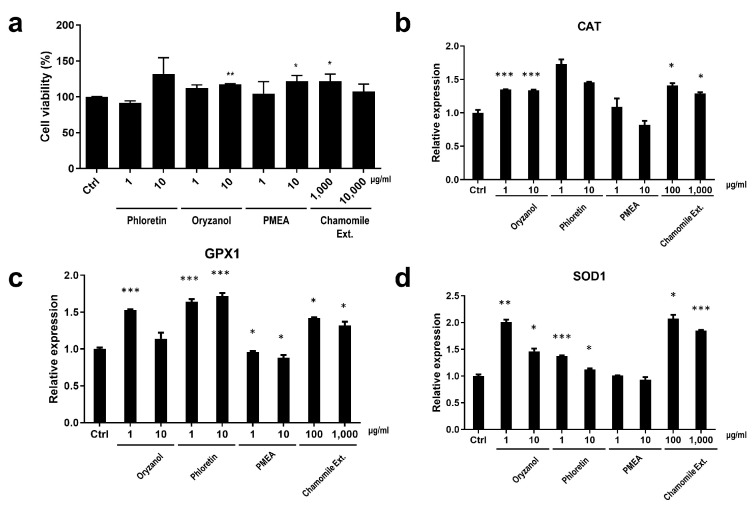
Analysis of the anti-oxidant effects of *BNC2* expression-regulating materials through in vitro experiments. (**a**) Analysis of Hs68 cell viability of *BNC2* expression-downregulating materials. (**b**) The enhancement of *CAT* expression by *BNC2*-downregulating materials. (**c**) The enhancement of *GPX1* expression by *BNC2*-downregulating materials. (**d**) The enhancement of *SOD1* expression by *BNC2*-downregulating materials. * *p* < 0.05, ** *p* < 0.01, *** *p* < 0.001; student *t*-test.

As the downregulation of *BNC2* expression reduced H_2_O_2_-induced cell death, we investigated whether materials that decreased *BNC2* expression could affect cell viability. In Hs68 cells treated with 500 μM H_2_O_2_ to induce cell death, we observed an increase in cell viability after treatment with the materials that decreased *BNC2* expression. A significant increase in cell viability was observed after treatment with oryzanol (10 μg/mL, *p* < 0.01), PMEA (10 μg/mL, *p* < 0.05), and chamomile extract (1000 μg/mL, *p* < 0.05) (Figure 5a). Although we observed the highest cell viability in the phloretin-treated (10 μg/mL) group, it was not significant (Figure 5a).

Anti-oxidant systems in the body play a crucial role in protecting cells from damage caused by harmful molecules such as free radicals, including ROS [51]. An important anti-oxidant system includes the enzymes catalase (CAT), superoxide dismutase (SOD), and glutathione peroxidase (GPX), and they work together to neutralize and remove harmful free radicals and peroxides [52,53,54]. By maintaining a balance between the production and elimination of ROS, this antioxidant system helps protect cells from oxidative stress and maintain their overall health and function. Therefore, we assessed whether the *BNC2*-downregulating materials activate the anti-oxidant system-related gene expression.

The expression of *CAT* was significantly increased by oryzanol (*p* < 0.001) and chamomile extract (*p* < 0.05) (Figure 5b). Although phloretin and PMEA promoted the expression of *CAT*, the increase was not statistically significant (Figure 5b). *GPX1* expression was significantly promoted by oryzanol (*p* < 0.001), phloretin (*p* < 0.001), and chamomile extract (*p* < 0.05), while PMEA (*p* < 0.05) caused a minimal enhancement in *GPX1* expression (Figure 5c). *SOD1* expression was significantly increased by oryzanol (*p* < 0.01) and chamomile extract (*p* < 0.05), followed by phloretin (*p* < 0.001) (Figure 5d). PMEA’s effect on the expression of *SOD1*, similar to that of *CAT* and *GPX1*, was found to be minimal (Figure 5d). Although the regulation of *CAT*, *GPX1*, and *SOD1* expression differed based on the treatment material, gene expression was mainly promoted by oryzanol, phloretin, and chamomile extract. These materials possibly activate the anti-oxidant system in the event of ROS occurrence, thereby increasing the cell survival rate; therefore, they may be considered remarkable cosmetic materials that can be used without concerns such as survival of the damage-accumulated cells.

In conclusion, we confirmed that materials that effectively decrease the expression of *BNC2* have anti-oxidant effects in dermal cells. Thus, we showed that these materials have the potential to be skin-improving ingredients that exert their anti-oxidant efficacy throughout the dermal layer.

### 3.4. Collagen Enhancement and Fine Wrinkle Improvement by an LG Formula Containing EDAR and BNC2 Expression-Regulating Materials

Our in vitro experiments revealed that lupeol, sucralfate, and cordycepin increase the expression of *EDAR* while promoting wound healing and epidermal and dermal gene regulation and that oryzanol, phloretin, and chamomile extracts promote anti-oxidant system-related genes while reducing the expression *BNC2*. Because ingredients with effective skin regeneration or anti-oxidation system activation ability can help improve wrinkles, we verified the wrinkle improvement effect of these materials in humans. We first confirmed their efficacy through a 3D skin model and then investigated the effect of improving fine wrinkles that appear around or under the eyes. We selected two ingredients for each target gene that exhibited a particularly remarkable overall efficacy to create an in-house LG H & H formula (LG formula) and analyzed its effectiveness through a 3D skin model and human clinical trial.

**Figure 6:**
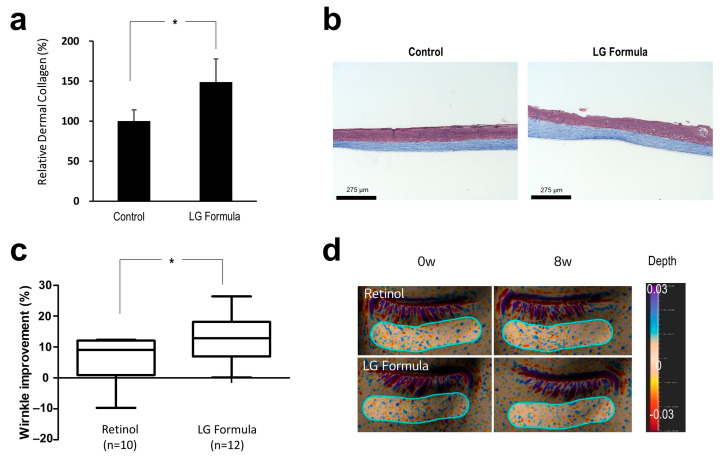
Dermal collagen enhancement and wrinkle improvement by the LG formula containing *EDAR* and *BNC2* expression-regulating materials. (**a**) The enhancement of dermal collagen by the LG formula. (**b**) Representative images of 3D skin; scale bar = 275 μm. (**c**) The comparison of the wrinkle improvement rate between retinol- and LG formula-treated groups. The LG formula included lupeol, sucralfate, oryzanol, and phloretin. (**d**) Representative images captured using Antera 3D at 0 and 8 weeks of treatment. * *p* < 0.05; student *t*-test.

To investigate whether the LG formula effectively enhances the dermal collagen content, we topically applied the LG formula onto the 3D skin model. Then, the 3D skin was sectioned and stained by Masson’s trichrome staining. We confirmed that the LG formula significantly enhances the dermal collagen content compared to the control formula (Figure 6a,b).

Next, we investigated the efficacy of the LG formula in improving fine wrinkles in vivo. A total of 6 female and 6 male volunteers with a mean age of 33 years (min. 27; max. 47) were included in the study. We then evaluated the efficacy of the LG formula compared to that of 0.1% retinol cream with the same base formulation using a half-test method. The changes in their under-eye wrinkles were assessed by topographic skin measurements using Antera 3D. We selected the Ra value of texture, which is a widely used indicator for the fine wrinkle measurement parameter [55]. The results of retinol application in two subjects were excluded from the analysis due to irritation on the applied area.

After 4 weeks of application, all measured wrinkles were slightly improved compared to the baseline value. However, after 8 weeks of application, the wrinkle improvement rate was significantly increased in both groups (Figure 6c). In particular, we discovered that the wrinkle improvement rate was significantly higher for the LG formula than for retinol (*p* < 0.05) (Figure 6c,d). Retinol has the disadvantage of causing consumer discomfort owing to skin irritation, such as burning and redness, despite its excellent efficacy; therefore, the LG formula may be used as a wrinkle-improving material with the same efficacy as retinol or better but without concerns about skin irritation. Further studies are needed to evaluate the effectiveness of the long-term use of LG formulas and their effects on other skin characteristics, including deep wrinkles, barrier function, and complexion changes. Furthermore, considering recent trends such as minimalism and eco-friendliness, additional research is needed to analyze the effectiveness of the individual ingredient alone regarding wrinkle improvement.

## 4. Conclusions

In the present study, we focused on two genes that have SNPs related to under-eye wrinkles and crow’s feet: EDAR related to wound-healing mechanisms and BNC2 related to cell reactivity to oxidative stress. We confirmed their functions in skin cells, namely, keratinocytes and fibroblasts, through gene knock-down experiments. With the decrease in *EDAR* expression, the wound-healing effect and the expression of wound healing-related genes decreased. With the decrease in *BNC2* expression, cell survival after H_2_O_2_ treatment and the expression of extracellular matrix-related genes increased. Based on these findings, we identified materials that regulate the expression of *EDAR* and *BNC2* and developed an LG formula that contained four ingredients with remarkable efficacy. Finally, we confirmed the outstanding effectiveness of the LG formula in improving wrinkles. Thus, we successfully developed a special anti-wrinkle formula containing active ingredients that regulate target genes, which were selected based on GWAS and mechanistic studies.

A limitation of the present study is that it evaluated the efficacy of materials based on the regulation of gene expression alone. Further in-depth research on each gene, such as identifying the amino-acid change due to the SNPs and the resultant altered proteins, will provide more direct solutions depending on the cause of aging.

We revealed the functions of periorbital wrinkle-related genes revealed in previous GWAS studies and showed the possibility of improving facial wrinkles by targeting these genes. This study suggests that two genes, *EDAR* and *BNC2*, may be possible targets for alleviating wrinkle formation and slowing down the aging process, and they provide a basis for developing anti-wrinkle products in the future.

## Figures and Tables

**Table 1:** List of active ingredients and their concentrations.

Active Ingredients	Concentration
Oryzanol	10 μg/mL
Phloretin	10 μg/mL
Cordycepin	0.1 μg/mL
Lupeol	10 μg/mL
Teprenone	10 μg/mL
Escin	10 μg/mL
Sucralfate	10 μg/mL
Madecassoside	10 μg/mL
Palmitamide MEA	10 μg/mL
Chamomile extract	1000 μg/mL
Calcium pantothenate	0.01 μg/mL
Panthenol	1 μg/mL
Troxerutin	1 μg/mL
Retinol	10 μM
Matrixyl-3000	1000 μg/mL
Peptilium	0.1 μg/mL
Polydatin	0.5 μg/mL
Peptilium	0.1 μg/mL
Bakuchiol	20 μM
Panax ginseng root protoplasts	100 μg/mL

## Data Availability

The data that support the findings of this study are available from the corresponding author on request.

## Supplementary Materials

The following supporting information can be downloaded at: https://www.mdpi.com/article/10.3390/biom14030279/s1, Figure S1: The screening of *EDAR* and *BNC2* expression-regulating materials among substances reported to have wrinkle improvement effects.
