# Supplementary material for: Fine Wrinkle Improvement through Bioactive Materials That Modulate EDAR and BNC2 Gene Expression
Source: Biomolecules. 2024 Feb 26;14(3):279. doi: 10.3390/biom14030279 (PMC10968300; doi:10.3390/biom14030279)
Supplement: Supplementary file 1 [file biomolecules-14-00279-s001.zip › biomolecules-2877864-supplementary.pdf]

biomolecules

MDPI

Supplementary Material: Fine Wrinkle Improvement through Bioactive Materials that Modulate *EDAR* and *BNC2* Gene Ex-pression

Seonju Lee, Sanghyun Ye, Mina Kim, Hyejin Lee, Seung-Hyun Jun * and Nae-Gyu Kang *

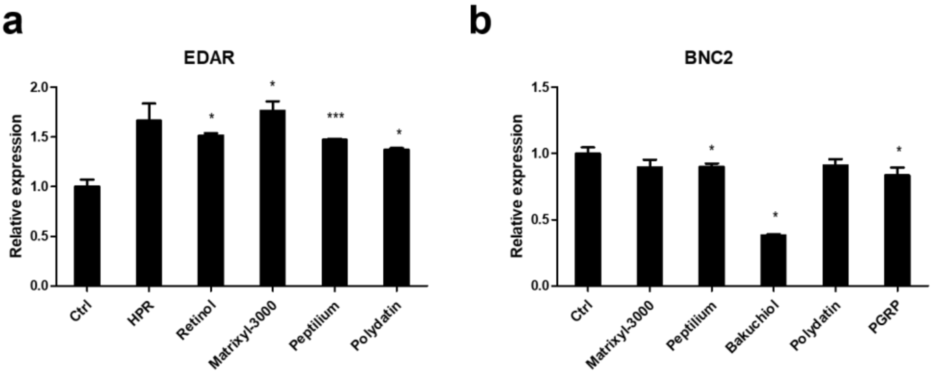

Figure S1. The effect of wrinkle improving materials on *EDAR* and *BNC2* expression. **(a)** The materials that increased the expression of *EDAR*. Relative expression of *EDAR* in keratinocytes (HaCaT) treated with various candidates. **(b)** The materials that decreased the expression of *BNC2*. Relative expression of *BNC2* in fibroblasts (Hs68) treated with various candidates. Error bars indicate standard error of the mean. *$p < 0.05$, ***$p < 0.001$; Student's t-test.
